# The association between urinary kidney injury molecule 1 and urinary cadmium in elderly during long-term, low-dose cadmium exposure: a pilot study

**DOI:** 10.1186/1476-069X-10-77

**Published:** 2011-09-05

**Authors:** Valérie Pennemans, Liesbeth M De Winter, Elke Munters, Tim S Nawrot, Emmy Van Kerkhove, Jean-Michel Rigo, Carmen Reynders, Harrie Dewitte, Robert Carleer, Joris Penders, Quirine Swennen

**Affiliations:** 1 Biomedical Research Institute, Hasselt University and transnational University Limburg, School of Life Sciences, Diepenbeek, Belgium; 2 Centre for Environmental Sciences, Hasselt University and transnational University Limburg, School of Life Sciences, Diepenbeek, Belgium; 3 Occupational & Environmental Medicine, Leuven University (KULeuven), Leuven, Belgium; 4 Department of Clinical Biology, Ziekenhuis Oost-Limburg (ZOL), Genk, Belgium; 5 Department of General Practice, Leuven University (KULeuven), Leuven, Belgium; 6 Primary health care center GVHV, Genk, Belgium

**Keywords:** kidney injury molecule 1, cadmium, renal biomarkers, toxicity, chronic, kidney

## Abstract

**Background:**

Urinary kidney injury molecule 1 is a recently discovered early biomarker for renal damage that has been proven to be correlated to urinary cadmium in rats. However, so far the association between urinary cadmium and kidney injury molecule 1 in humans after long-term, low-dose cadmium exposure has not been studied.

**Methods:**

We collected urine and blood samples from 153 non-smoking men and women aged 60+, living in an area with moderate cadmium pollution from a non-ferrous metal plant for a significant period. Urinary cadmium and urinary kidney injury molecule 1 as well as other renal biomarkers (alpha1-microglobulin, beta2-microglobulin, blood urea nitrogen, urinary proteins and microalbumin) were assessed.

**Results:**

Both before (r = 0.20; p = 0.01) and after (partial r = 0.32; p < 0.0001) adjustment for creatinine, age, sex, past smoking, socio-economic status and body mass index, urinary kidney injury molecule 1 correlated with urinary cadmium concentrations. No significant association was found between the other studied renal biomarkers and urinary cadmium.

**Conclusions:**

We showed that urinary kidney injury molecule 1 levels are positively correlated with urinary cadmium concentration in an elderly population after long-term, low-dose exposure to cadmium, while other classical markers do not show an association. Therefore, urinary kidney injury molecule 1 might be considered as a biomarker for early-stage metal-induced kidney injury by cadmium.

## Background

Cadmium (Cd) is an ever-present and global environmental pollutant [1]. Current Cd emission has been drastically reduced, but Cd continues to be a health hazard, because historically accumulated Cd cannot be degraded and its half-life in the body is long (10-30 years) [2]. Next to the bone [3], a main target for chronic, low-level Cd exposure is the kidney, leading to proximal tubule dysfunction [2,4,5]. Tubular dysfunction causes polyuria and low molecular weight proteinuria and some of these urinary proteins, e.g. α-1 microglobulin (α1M-U)[6], β-2 microglobulin (β2M-U)[7,8] and clara cell protein-16 (CC-16-U)[9] are used as urinary biomarkers of the early stages of Cd nephrotoxicity. In other studies, Cd toxicity is monitored by the Cd-binding protein metallothionein in urine [8,10,11], N-acetyl-beta-glucosaminidase (NAG) [7,8] or even urinary Cd itself [7,11-13]. Although these renal biomarkers are widely used, questions arose concerning specificity and sensitivity [6,9,11,14-17]. There clearly is a need for an early and stable biomarker for proximal tubule damage caused by Cd.

Kidney injury molecule 1 (KIM-1), originally discovered by Ichimura et al., is a type 1 membrane glycoprotein found on renal proximal tubule epithelial cells. It contains in its extracellular portion a unique 6-cysteine immunoglobulin-like domain and a mucin-domain [18]. An intracellular highly conserved tyrosine kinase phosphorylation motif is a strong indicator that KIM-1 is a cell signaling molecule [19].

KIM-1 expression is induced in a variety of renal diseases, whereas in healthy kidney tissue KIM-1 is virtually undetectable [18,20]. In the case of kidney damage, KIM-1 is expressed on the apical membrane followed by cleavage of the ectodomain (90 kDa) which is released in the urine in rodents [18,19,21-24] and in humans [25-28]. KIM-1 is upregulated in the proximal tubule during dedifferentiation of the kidney epithelium, an early manifestation in response to damage [29].

In rats, Prozialeck et al. showed that KIM-1 is a very early urinary marker for Cd-induced kidney injury [23]. They showed that KIM-1 was elevated before other urinary biomarkers of Cd nephrotoxicity, such as metallothionein, CC-16-U, proteinuria, α-glutathione-S-transferase (α-GST), NAG and Cd itself [23,30]. Moreover they showed that the Cd-induced increase in KIM-1 expression can be detected before signs of necrosis appear and when there is only a modest level of apoptosis in the proximal tubule [29].

It has been well established in humans that KIM-1 appears in the urine at an early stage in kidney damage and also, that Cd affects proximal tubule function when the Cd burden is high. Cd triggers the expression of KIM-1 at a very early stage in animal models [31]. The aim of the present pilot study is to assess the appearance of urinary KIM-1 after long-term, low-dose Cd exposure in humans, because, to our knowledge, this has not been investigated in the population.

## Methods

### Study population and sample collection

The total population (n = 3069) of the general practice in Genk is registered in the framework of a registration network for family practices in Flanders (INTEGO) [32]. The study area is representative of the total population. Non-smoking men and women, 60 to 80 years old, with no acute infection at enrolment and no history of malignancies, were selected in the southern region of Genk from a quarter adjacent to an industrial area where a non-ferrous metal plant, a major motor company and a power station are located and which is crossed by multiple highroads. Sampling was combined with the annual influenza vaccination at a local doctor's practice. Eligible people were notified in advance by letter. Of those that routinely are advised for influenza vaccination, approximately 86% joined the vaccination program. Of those that were eligible, 154 were recruited, and 99% agreed to participate in our study. Of the 153 persons that agreed to participate (79 women; mean age 71 yr and 74 men; mean age 70 yr), for one person no urinary sample was collected and this person was not included in the analyses. Personal information was processed anonymously in conformity with privacy policy. Informed consent was obtained from all participants and the study was approved by the ethics committee of the Ziekenhuis Oost-Limburg (ZOL), Belgium. Questionnaires were administered to assess lifestyle, profession, education, past smoking status, as well as data on age, weight and gender. Family income was given as net monthly overall family income and subdivided into low (<1500€), medium (1500€ - 3000€) and high (>3000€) family income. Education was coded as low (primary school), medium (high school) and high (university). Past smoking was quantified as pack years by multiplying the number of packs of cigarettes smoked per day by the number of years the person has smoked. Finally, individual medical backgrounds were used to determine possible interference of drug administration or diseases, with kidney function. Second morning urine samples and blood samples were collected from all participants. Samples were aliquoted (6 × 2 ml), stored on ice for a maximum of four hours and subsequently frozen at -80°C.

### Routine analyses and renal biomarker measurements

Routine analyses of the urine samples were performed in the clinical laboratory of the regional hospital ZOL in Genk. Using an automated analyzer (Modular^® ^P800-ISE900 System, Roche Diagnostics; Mannheim, Germany), the following urinary analyses were performed, according to manufacturer's instructions: creatinine according to the kinetic Jaffe method (compensated, rate blanked), total protein by a colorimetric biuret test and α1M-U based on immunological agglutination. β2M-U was determined by particle-enhanced immunonephelometry using the BN ProSpec (Siemens Healthcare Diagnostics; Marburg, Germany). Microalbumin was nephelometrically determined (Immage^® ^Immunochemistry System, Beckman Coulter; Suarlee, Belgium). Blood urea and serum creatinine were measured following the same assays as with the urinary analyses. BUN (blood urea nitrogen) was determined as blood urea times two (covering the molar mass of the two nitrogens). Urinary KIM-1 was analyzed by a commercially available sandwich ELISA: Human TIM-1/KIM-1/HAVCR Duoset (R&D Systems; Abingdon, U.K.), validated by Chaturvedi et al. [33]. The assay procedure was performed according to the prescriptions of the manufacturer. When necessary, samples were adjusted to pH 7.0 before measurement [34]. The optical density was determined with a fluorescence microplate reader (FLUOstar OPTIMA, BMG Labtechnologies; Offenburg, Germany), set to 450 nm with a wavelength correction at 540 nm. All samples were measured in duplicate.

### Urinary Cd analyses

Cd concentrations in urine were analyzed by means of inductively coupled plasma mass spectrometry (ICP-MS) using the ELAN^® ^DRC-e (Axial Field™ Technology, Perkin Elmer SCIEX; Zaventem, Belgium). Urine samples and standards were diluted 1:10 in 1% nitric acid.

### Statistical analyses

For database management and statistical analyses, SAS Software version 9.1 (version 9.1, SAS Institute Inc, Cary (NC), USA) and GraphPad Prism 5.01 (GraphPad Software Inc, La Jolla (CA), USA) were used. Non-normally distributed data were log transformed. For comparison of means and proportions, we applied Student's t-test and the χ^2^-statistic, respectively. We investigated associations between markers of kidney function and urinary cadmium using Pearson's correlation and multiple linear regression. Estimated effect sizes and 95% CI were calculated from linear regression coefficients for a two-fold increase in urinary Cd. A priori three models were chosen: model 1 shows unadjusted data, in model 2, results are adjusted for creatinine, sex, age, past smoking, body mass index (BMI) and socio-economic status (SES; based on educational degree and monthly family income), while in model 3 data are adjusted for sex, age, past smoking, BMI and SES and given as function of creatinine. When residuals are calculated (figures), we adjusted the different parameters for creatinine, sex, age, past smoking, BMI and SES, in order to remove these potential confounding factors from the association. Correlations were considered significant when p < 0.05. All tests were two-sided.

## Results

The study population consists of 153 participants (52% women) with a mean age of 71 years. Patient characteristics can be found in table 1. From all the participants, of the 54% that have ever smoked there was a significant difference between men and women (75% and 35% respectively, p < 0.0001). Those who had smoked in the past had an average of 18 pack years. The average distance between their residence and the heavy metal industrial zone was 2743 m, while the mean distance to the two main roads was 294 m and 562 m. Participants have lived at their current addresses for a mean period of 36 years (range: 3 to 75 years). Geometric mean urinary Cd level was 0.76 μg/g creatinine. Geometric mean of the urinary KIM-1 concentrations as well as the mean concentrations of other renal biomarkers (β2M-U, α1M-U, BUN, urinary proteins, microalbuminuria) and creatinine are given in table 2.

**Table 1:** Participants characteristics

Characteristics	Total group (n = 153)
Anthropometrics	
Sex, female	79 (52%)
Age, years	71 ± 4.5
BMI, kg/m^2^	27.2 ± 4.3
Socio-economic status*°	
Low	62 (41%)
Median	59 (39%)
High	29 (19%)
Familial income, per month°	
<1500€	71 (47%)
1500-3000€	77 (51%)
>3000€	2 (1%)
Smoking status	
Ex-smoker^†^	81 (54%)
Never smoked^†^	68 (46%)
Exposure to environmental tobacco smoke^¶^	61 (48%)
Use of medication^§^	
Antiplatelet medication	13 (9%)
Statins	81 (53%)
ACE inhibitor	27 (18%)
Insulin	5 (3%)
Antidiabetic medication	19 (13%)
NSAID	23 (15%)
Blood analyses	
Hemoglobin, g/dl	14.17 ± 1.24
Red blood cells, 10^6^/μl^"^	4.75 (4.68 - 4.82)
White blood cells, 10^3^/μl^"^	6.58 (5.76 - 7.53)
Neutrophils, %^"^	55.10 (53.60 - 56.66)
Lymphocytes, %^"^	29.70 (28.35 - 31.12)
Monocytes, %^"^	6.52 (5.76 - 7.53)
Eosinophils, %^"^	2.79 (2.39 - 3.25)
Ferritin, ng/ml^"^	121.9 (108.0 - 137.6)
CRP, mg/dl	
Creatinine, mg/dl^"^	0.86 (0.83 - 0.89)
Glucose, mg/dl^"^	99.95 (96.50 - 103.50)
Cholesterol, mg/dl	200.7 ± 37.26
HDL, mg/dl^"^	56.64 (54.04 - 59.37)
LDL, mg/dl	114.4 ± 34.48
Triglycerides, mg/dl^"^	122.1 (112.8 - 132.1)

Data are arithmetic mean ± standard deviation or absolute number (percentage of study population);

^" ^data which are not normally distributed and for which geometric mean (95% confidence interval) is given.

*Based on educational degree; °n = 150; ^†^n = 149; ^§^n = 152;^¶ ^n = 126

**Table 2:** Mean urinary cadmium and renal biomarker values

	Mean (95% CI)
Cadmium, μg/l†	0.80 (0.73 - 0.88)
Cadmium/creatinine, μg/g creat†	0.76 (0.70 - 0.84)
	
KIM-1, pg/ml*	569 (498 - 651)
KIM-1/creatinine, μg/g creat*	0.55 (0.49 - 0.62)
	
α1-microglobulin, mg/l†	3.19 (2.58 - 3.91)
α1-microglobulin/creatinine, mg/g creat†	2.97 (2.42 - 3.64)
	
Proteins, mg/l*	71.80 (63.81 - 80.80)
Proteins/creatinine, mg/g creat*	66.61 (59.99 - 73.95)
	
Albumin, mg/dl*	8.73 (7.46 - 10.21)
Albumin/creatinine, mg/g creat*	8.43 (7.32 - 9.70)
	
β2-microglobulin, mg/l‡	0.12 (0.11 - 0.13)
β2-microglobulin/creatinine, mg/g creat‡	0.12 (0.11 - 0.13)
	
BUN, mg/dl†	35.94 (34.47 - 37.46)
Urinary creatinine, mg/dl*	104.9 (96.55 - 114.0)

Geometric mean (95% confidence interval) of urinary cadmium and renal biomarkers. All parameters were measured in urine; except for blood urea nitrogen, which was measured in blood. † n = 152; * n = 153; ‡ n = 140.

**Figure 1:**
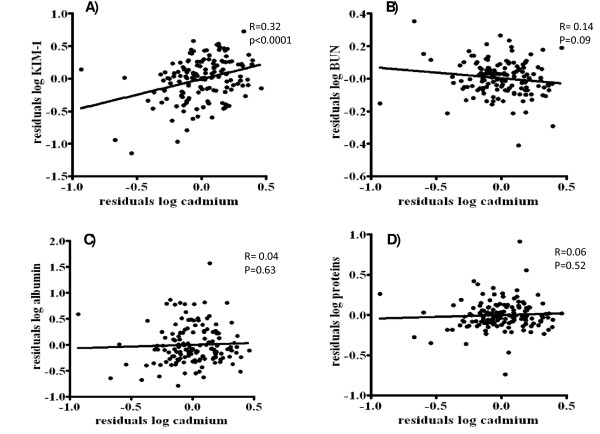
**Correlations of the residuals of urinary KIM-1 and the other biomarkers with the residuals of urinary cadmium**. Correlations of the residuals of A) log KIM-1, B) log blood urea nitrogen, C) log albumin and D) log proteins with the residuals of log cadmium. All parameters were measured in urine, except for blood urea nitrogen. Residuals were computed to remove the variance by age, sex, socio-economic status, body mass index, creatinine (except for blood urea nitrogen) and past smoking. Data on KIM-1, cadmium, protein, blood urea nitrogen and albumin concentrations were log-transformed to obtain a normal distribution. Abbreviations:KIM-1: kidney injury molecule 1; BUN, blood urea nitrogen.

Urinary KIM-1 was not influenced by gender (p = 0.83), age (p = 0.08), distance between housing and industrial zone (p = 0.57), SES (p = 0.40), past smoking (p = 0.14) and BMI (p = 0.83). Both before (table 3 and Figure 1) and after adjustment (table 3) for sex, age, past smoking, BMI and socio-economic status (including education and income) variables, KIM-1 correlated positively and significantly with the urinary cadmium concentration. For the other biomarkers (BUN, microalbuminuria and urinary proteins) unadjusted and adjusted multiple linear regression models showed no significant correlation between these biomarkers of kidney function and urinary Cd (see Figure 1 and table 3). Both for β2M-U and α1M-U a considerable amount of urine samples that were tested (84% and 28% respectively) were below the limit of detection, suggesting the assessments that were used were not sensitive enough. Therefore, no analysis was conducted for β2M-U and α1M-U in association with Cd.

**Table 3:** Estimated change (%) in urinary biomarker levels calculated for a two-fold increase in urinary cadmium concentration

	Estimated effect size (%)	95%CI(%)	R^2^	p-value
**Model 1**				
KIM-1	23.73	6.92 to 43.18	0.05	0.005
Microalbumin	4.40	-13.42 to 25.89	0.001	0.65
Proteins	5.66	-3.52 to 15.71	0.01	0.24
BUN	-5.65	-10.53 to -0.49	0.04	0.03
**Model 2**				
KIM-1	39.54	18.26 to 64.65	0.27	0.0001
Microalbumin	4.85	-14.14 to 28.03	0.27	0.64
Proteins	3.15	-6.26 to 13.52	0.49	0.53
BUN	-4.66	-9.83 to 0.80	0.08	0.10
**Model 3**				
KIM-1	26.59	7.82 to 48.62	0.13	0.005
Microalbumin	-2.80	-20.42 to 18.78	0.09	0.78
Proteins	0.66	-8.65 to 10.92	0.11	0.89
BUN	-3.69	-8.99 to 1.91	0.07	0.19

All data were log-transformed and estimated effect size is given as % estimated effect size calculated for a two-fold increase in urinary cadmium concentrations (μg/g creatinine) with the corresponding 95% confidence interval, R^2 ^gives the explained variance.

Abbreviations: KIM-1, kidney injury molecule 1; BUN, blood urea nitrogen; CI, confidence interval

Model 1: unadjusted but given as function of creatinine

Model 2: adjusted for creatinine (except for blood urea nitrogen), sex, age, past smoking, body mass index and socio-economic status

Model 3: adjusted for sex, age, past smoking, body mass index and socio-economic status; given as a function of creatinine.

## Discussion

About a decade ago, KIM-1 was discovered in the search for molecules involved in the pathogenesis of acute kidney injury. We demonstrated among elderly a robust association between urinary KIM-1 and urinary Cd. Depending on the biomarker of nephrotoxicity thresholds of urinary Cd can range from about 2.4 μg Cd/g creatinine for the onset of early biochemical alterations (e.g. hypercalciuria) to 10 μg Cd/g creatinine for the development of the classic tubular microproteinuria [13]. Here, we showed biochemical changes at urinary Cd levels below 1 μg Cd/g creatinine.

Ichimura et al. were the first to describe KIM-1 as a type 1 membrane glycoprotein, which contains a 6-cystein immunoglobulin-like domain in its extracellular portion, and a Thr/Ser-Pro rich domain characteristic of mucin-like O-glycosylated proteins [18]. KIM-1 also has a transmembrane domain and a cytoplasmic domain, which contains a conservative tyrosine kinase phosphorylation site, indicating that KIM-1 may be a signaling molecule [19]. In healthy kidney tissue, KIM-1 is virtually undetectable whereas in the injured kidney, KIM-1 expression is rapidly upregulated at the apical side of the proximal tubule [18,21]. This process is accompanied by the shedding of the extracellular domain of KIM-1 into the urine [19]. The ectodomain is stable in urine [34] and has shown to be a sensitive biomarker of renal injury induced by a variety of agents including the heavy metal Cd: Prozialeck et al. have proven KIM-1 to be a putative early biomarker for proximal tubule damage caused by high doses of Cd in rats, outperforming the classic biomarkers such as metallothionein, CC-16, α-GST and urinary proteins [23,29,30]. In addition, KIM-1 appears before any lethal injury is detected in the proximal tubule epithelial cells [29]. Thus, using this biomarker, very early detection of cell stress may be possible, which would allow for the reversal and/or the treatment of Cd-induced kidney injury [35]. To our knowledge however, no research has ever focused on the correlation of the Cd burden and the urinary KIM-1 concentrations in humans.

Since we were mainly interested in the possible role for KIM-1 as a biomarker after long term environmental Cd intoxication, we chose a non-smoking elderly population living near a non-ferrous metal industrial zone in Genk for a longer period. In this region, levels of Cd have been reported to be higher than in other Belgian gauging regions according to the Flemish environmental agency (VMM: Mira-T indicator rapport 2008).

Both blood and urinary Cd are indicators of Cd body burden; however urinary Cd correlates better with the duration of exposure than does blood Cd [12,36,37], which makes it a better indicator for long-term Cd exposure. Therefore, we compared the urinary Cd concentrations with KIM-1 and other biomarkers of nephrotoxicity.

As shown in Figure 1, after adjustment for creatinine, sex, age, past smoking, BMI and SES, only KIM-1 was significantly correlated with Cd levels.

Urinary Cd concentration averaged below 1 μg/g creatinine among our population. This may explain why albuminuria, proteinuria and BUN did not correlate with urinary Cd. These markers mainly identify later stages of Cd-induced kidney injury [38,39].

Although α1M-U is stable across physiological pH [40], its specificity is undermined by the influence of several conditions such as liver disease [15], HIV [16], mood disorders [17] and other environmental influences, for example lead exposure [41]. In contrast to β2M-U, which is degraded rapidly in acidic urine [42], urinary KIM-1 has been proven to be stable over the physiological range of urinary pH values [34]. Moreover, it originates from proximal tubule cells [20,43], which makes it a much more specific renal biomarker than proteins originating from other parts of the body. Prozialeck et al. found in rats that urinary KIM-1 starts to increase significantly earlier and at lower doses of Cd than metallothionein, CC-16, proteins and α-GST [29,30,44]. The present study corroborates this high sensitivity for human subjects.

Since KIM-1 has a high cysteine content and Cd is known for its high binding capacity with cystein complexes [45], future research should focus on whether this influences the correlation between urinary KIM-1 and urinary Cd.

## Conclusions

In conclusion, this pilot study shows that urinary KIM-1 levels are significantly correlated with urinary Cd levels in an elderly population after long-term, low-dose exposure to Cd, probably indicating beginning metal-induced kidney injury. To further elucidate the exact role KIM-1 can play as a biomarker for early cadmium-induced renal damage, future research should concentrate on the comparison of KIM-1 with other biomarkers by using the most sensitive and reliable measuring techniques, with inclusion of a paired control group, living in unpolluted areas.

## List of abbreviations

Cd: cadmium; α1M-U: urinary α-1 microglobulin; α-GST: α-glutathione-S-transferase; β2M-U: urinary β-2 microglobulin; BMI: body mass index; BUN: blood urea nitrogen; CC-16-U: urinary clara cell protein-16; CI: confidence interval; ICP-MS: inductively coupled plasma mass spectrometry; KIM-1: kidney injury molecule 1; NAG: N-acetyl-beta-glucosaminidase; SES: socio-economic status; ZOL: Ziekenhuis Oost-Limburg.

## Competing interests

The authors declare that they have no competing interests.

## Authors' contributions

VP participated in the design of the study, the sample collection, KIM-1 measurements, the analyses of the data and the writing of the manuscript. LMD was involved in the sample collection and the KIM-1 measurements. EM participated in the design of the study and the sample collection. TN was involved in the design of the study and conducted the statistical analyses. EV and JR participated in the design of the study. CR carried out the routine analyses. HDW was involved in the design of the study and the sample collection. RC carried out the urinary cadmium analyses. JP and QS were both involved in the design of the study and the data analyses. All authors read and approved the final manuscript.
